# Candidate Biological Indicators of Occupational Stress in Healthcare Workers: Heart Rate, Free Triiodothyronine and HDL Cholesterol—A Cross-Sectional Study

**DOI:** 10.3390/nursrep16080277

**Published:** 2026-08-07

**Authors:** Tanja Lupieri, Martina Hrvačić, Dubravka Švob Štrac, Suzana Uzun, Dunja Degmečić

**Affiliations:** 1 Clinical Hospital “Sveti Duh”, 10000 Zagreb, Croatia; mhrvacic@kbsd.hr; 2 Laboratory for Molecular Neuropsychiatry, Division of Molecular Medicine, Ruđer Bošković Institute, 10000 Zagreb, Croatia; dsvob@irb.hr; 3 Department of Biological Psychiatry and Psychogeriatrics, University Psychiatric Hospital Vrapče, 10000 Zagreb, Croatia; suzana.uzun@gmail.com; 4 School of Medicine, University of Zagreb, 10000 Zagreb, Croatia; 5 Department of Psychiatry, University Hospital Centre Osijek, 31000 Osijek, Croatia; dunja.degmecic@mefos.hr; 6 Faculty of Medicine Osijek, J. J. Strossmayer University of Osijek, 31000 Osijek, Croatia

**Keywords:** occupational stress, healthcare workers, biomarkers

## Abstract

**Background/Objectives:** Healthcare staff, especially nurses, face high occupational stress, yet objective biological indicators that complement self-report are not well established. We examined whether metabolic, hormonal and physiological markers act as indicators of stress and relate to anxiety, depression and burnout in nurses and other healthcare workers. **Methods:** We conducted a cross-sectional study of 300 hospital employees (239 in healthcare, of whom 123 were nurses; 61 non-healthcare) at a Croatian university hospital; of 329 completed questionnaires, 300 (91.2%) met the inclusion criteria and were analysed. Fasting glucose, lipids, thyroid hormones, cortisol, heart rate and blood pressure were measured; psychological status was measured using the 42-item Depression Anxiety Stress Scales (DASS-42), Maslach Burnout Inventory and Zung scale. Analyses used Mann–Whitney and Kruskal–Wallis tests, Spearman correlation, hierarchical logistic regression adjusted for age, sex, smoking and thyroid medication use, and receiver operating characteristic (ROC) analysis. **Results:** Healthcare workers had more favourable glucose, total and low-density lipoprotein (LDL) cholesterol than the older non-healthcare group (all *p* ≤ 0.003); because the groups differed substantially in age, these unadjusted differences are descriptive only. Metabolic markers correlated only weakly with distress and burnout. Within healthcare workers, elevated stress was discriminated in univariable analysis by low high-density lipoprotein (HDL) cholesterol (area under the curve (AUC) 0.72), raised triglycerides (AUC 0.67) and glucose (AUC 0.66); however, after adjustment for age, sex, smoking and thyroid medication use, none of the markers remained independently associated with elevated stress. **Conclusions:** In this relatively healthy hospital workforce, routinely available biological markers showed limited value as standalone indicators of occupational stress; they are exploratory rather than validated biomarkers, and require confirmation in larger, prospective, multicentre studies before they can complement—rather than replace—psychological screening in occupational-health practice.

## 1. Introduction

Occupational stress is common among healthcare staff, who face heavy workloads, shift work, emotional demands and exposure to suffering. Nurses, the largest segment of the hospital workforce, are particularly affected. Sustained exposure is associated with burnout, anxiety and depression, and a high burnout prevalence has been reported internationally and during the COVID-19 pandemic [1,2,3,4,5]. Stress is not only a psychological experience but also a physiological one: acute stressors activate the sympathetic nervous system and the hypothalamic–pituitary–adrenal (HPA) axis, while prolonged activation can dysregulate these systems [6,7,8,9]. A recognised consequence of chronic stress is disturbed metabolism. Cortisol promotes hepatic gluconeogenesis, mobilises lipids and, over time, favours insulin resistance and an atherogenic lipid profile. Prospective and clinical studies have linked burnout and work stress to a higher risk of type 2 diabetes, altered glucose homeostasis, raised triglycerides and reduced HDL cholesterol [10,11,12,13,14,15]. Work-related stress also clusters with metabolic syndrome and cardiovascular disease [16,17], and the hypothalamic–pituitary–thyroid axis is sensitive to chronic strain [18]. Despite these mechanisms, few studies have tested whether routinely available metabolic and physiological markers serve as objective indicators of occupational stress in nurses and other healthcare staff, or how they relate to anxiety and burnout. We therefore examined whether metabolic markers (glucose and lipids) and related biochemical and physiological measures (thyroid hormones, cortisol, heart rate) act as biological indicators of stress in healthcare staff and are associated with anxiety, depression and burnout. We paid particular attention to indicators less frequently examined together in a hospital occupational-health sample, namely resting heart rate and free triiodothyronine (fT3). The novelty of the present study lies in the simultaneous assessment of psychological stress, metabolic parameters, resting heart rate and thyroid hormones within a cohort of hospital healthcare workers undergoing occupational-health evaluation.

## 2. Materials and Methods

### 2.1. Study Design and Participants

**Figure 1:**
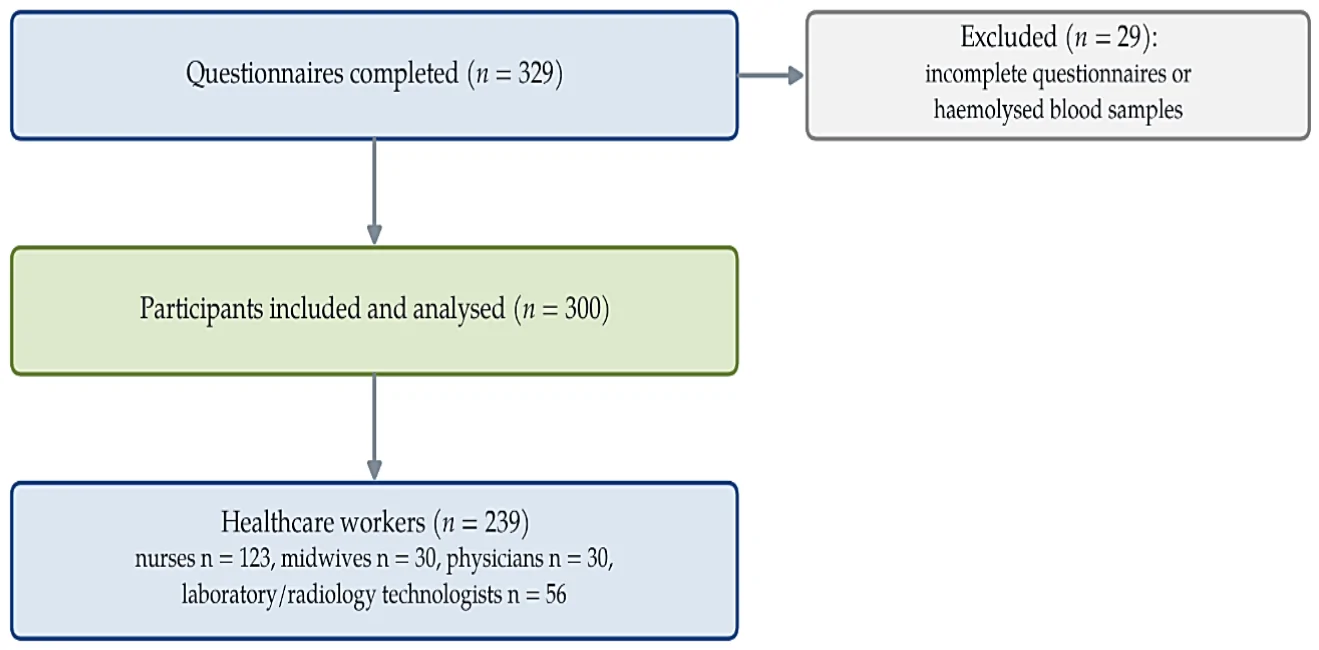
Participant flow diagram.

We conducted a cross-sectional study at University Hospital “Sveti Duh”, Zagreb, Croatia. The sample comprised 300 employees: 239 healthcare workers (physicians, nurses and medical technicians, midwives, physiotherapists, laboratory and radiology technologists, and pharmacists; nurses were the largest group, *n* = 123) and 61 non-healthcare workers (administrative, technical, service and catering staff, teachers, and individuals attending routine medical examinations) as a comparison group. Recruitment took place at the Day Hospital (Routine Medical Examination Clinic) of University Hospital “Sveti Duh” over an approximately three-month period between February and May 2025. Participation was voluntary and anonymous; data were collected with a structured, self-administered questionnaire distributed both electronically (Google Forms, via an emailed link) and on paper at the clinic, and recruitment was by open invitation rather than random sampling, so the sample is one of convenience. Inclusion criteria were age at least 18 years, active employment and written informed consent. We excluded participants with a severe mental disorder, acute or chronic illness likely to affect biological markers, haemolysed samples or incomplete questionnaires. Of 329 staff members who completed the questionnaire, 29 were excluded (incomplete questionnaires or haemolysed blood samples), leaving 300 participants (91.2%) for analysis: 239 healthcare workers and 61 non-healthcare comparison participants (Figure 1). Analyses were performed on complete cases only, and no missing values were imputed. No a priori sample-size or power calculation was performed; the achieved sample size was determined by staff availability during the recruitment period. The achieved sample size was determined by the number of staff available during the recruitment period; consequently, the healthcare and comparison groups were unequal in size, which reduces the precision of between-group estimates and limits statistical power for subgroup analyses.

### 2.2. Psychological Measures

Psychological status was assessed with validated Croatian versions of the Depression Anxiety Stress Scales (DASS-42) [19], the Maslach Burnout Inventory–Human Services Survey, measuring emotional exhaustion, depersonalisation and personal accomplishment [20], and the Zung Self-Rating Depression Scale [21]. Elevated stress was defined a priori from the DASS stress subscale as a score of at least 19 (moderate to extremely severe) versus 18 or below (normal to mild).

### 2.3. Physiological and Biochemical Measures

Venous blood was drawn once between 08:00 and 09:00 after at least 8 h of fasting and 30 min of seated rest and analysed at the hospital’s accredited laboratory. Glucose, total cholesterol, triglycerides, HDL cholesterol and LDL cholesterol (all mmol/L) were measured on an Alinity c analyser (Abbott Diagnostics, Lake Forest, IL, USA). Thyroid-stimulating hormone, free triiodothyronine (fT3), free thyroxine (pmol/L), cortisol (nmol/L) and progesterone (nmol/L) were measured by electrochemiluminescence immunoassay (Cobas e601, Roche Diagnostics, Mannheim, Germany). Resting heart rate (beats/min) and blood pressure (mmHg) were recorded with a validated automatic oscillometric device (Microlife BP A6 PC, Microlife AG, Widnau, Switzerland) once in the morning under standardised conditions (after the same 8 h fast and 30 min of seated rest), simultaneously.

### 2.4. Statistical Analysis

Normality of continuous variables was assessed with the Shapiro–Wilk test. Non-normally distributed data are presented as medians with interquartile ranges (IQRs). Comparisons between groups were performed using the Mann–Whitney U test, with the Hodges–Lehmann estimate of the difference in medians and 95% confidence intervals (CIs). The association of continuous variables was assessed by Spearman’s rank correlation coefficient. Categorical variables were compared using the chi-squared test.

In healthcare workers, a hierarchical multivariate logistic regression analysis was performed to identify factors associated with increased stress. Model 1 included age, gender, smoking status, and thyroid medication use as clinically relevant confounders. Model 2 additionally included heart rate, high-density lipoprotein (HDL) cholesterol, triglycerides, and free triiodothyronine (fT3) to assess their additional contribution. Multicollinearity was assessed using the variance inflation factor (VIF).

Confounders were selected a priori on clinical and theoretical grounds rather than by statistical significance. Age and sex were included because both are established determinants of lipid and metabolic profile, resting heart rate and thyroid-hormone concentrations, and are also associated with the reporting of psychological distress; failing to adjust for them could therefore bias the biomarker–stress associations. Smoking was included because it directly lowers HDL cholesterol and raises triglycerides and heart rate. Thyroid-medication use was included because it directly alters circulating thyroid hormones, including free triiodothyronine (fT3), which is one of the markers of interest. Variables were entered in a prespecified hierarchical sequence—demographic and clinical confounders in Model 1, and the biological markers (heart rate, HDL cholesterol, triglycerides and fT3) in Model 2—to keep the model parsimonious given the number of elevated-stress events and to assess the additional contribution of the biomarkers beyond the confounders.

Receiver operating characteristic (ROC) analysis was performed to assess the discriminatory ability of individual biomarkers for elevated stress. Area under the ROC curve (AUC), sensitivity, specificity, and exploratory cut-offs based on the Youden index are presented. The discriminatory performance of the final multivariate model was further assessed using the AUC calculated from the model-predicted probabilities. Model calibration was assessed using the Hosmer–Lemeshow goodness-of-fit test, and overall model performance was summarised using Nagelkerke’s R^2^. Since both the multivariate model and the ROC curve-derived cut-offs were developed and evaluated on the same dataset without internal or external validation, the findings should be considered exploratory.

All tests were two-sided, and the significance level was set at alpha = 0.05. Statistical analyses were performed using MedCalc version 23.4.8 (MedCalc Software Ltd., Ostend, Belgium; https://www.medcalc.org; accessed on 15 November 2025) [22]. Given the exploratory and hypothesis-generating nature of the analyses, no formal adjustment for multiple comparisons was applied. Consequently, nominal *p* values should be interpreted cautiously, particularly for isolated findings, and emphasis was placed on effect estimates, 95% CI and consistency across analyses.

## 3. Results

### 3.1. Sample Characteristics

Using DASS, at least moderate symptoms were reported for stress by 30.3% (95% CI 25.4–35.8) of participants, for anxiety by 97.7% (95% CI 95.3–98.9) and for depression by 95.3% (95% CI 92.3–97.2). On the Maslach Burnout Inventory, high emotional exhaustion was present in 39.3% (95% CI 34.0–45.0), high depersonalisation in 15.7% (95% CI 11.9–20.3) and high reduced personal accomplishment in 36.7% (95% CI 31.5–42.3). Healthcare workers were younger than non-healthcare workers (median 36 versus 47 years) and had shorter total work experience and current-post tenure (all *p* < 0.001); the sexes were similarly distributed (Table 1).

### 3.2. Metabolic and Biochemical Markers Between Groups

In the unadjusted comparison, healthcare workers had lower glucose, total cholesterol and LDL cholesterol concentrations than non-healthcare workers (all *p* ≤ 0.003). HDL cholesterol, triglycerides and cortisol did not differ significantly between the groups, whereas fT3 concentrations were slightly higher among healthcare workers. Because the groups differed substantially in age and other baseline characteristics, these findings should be interpreted as descriptive and should not be attributed solely to healthcare employment (Table 2).

### 3.3. Associations with Distress and Burnout

Across the whole sample, metabolic markers correlated weakly and mostly non-significantly with self-reported distress and burnout. The largest, though still non-significant, association was between glucose and depression (Spearman rho 0.11, *p* = 0.10), whereas HDL cholesterol was essentially uncorrelated with the DASS stress score (rho −0.04, *p* = 0.53). By contrast, heart rate—a physiological rather than metabolic measure—was correlated with emotional exhaustion (rho 0.20, *p* < 0.001).

### 3.4. Discrimination of Elevated Stress Within Healthcare Workers

**Figure 2:**
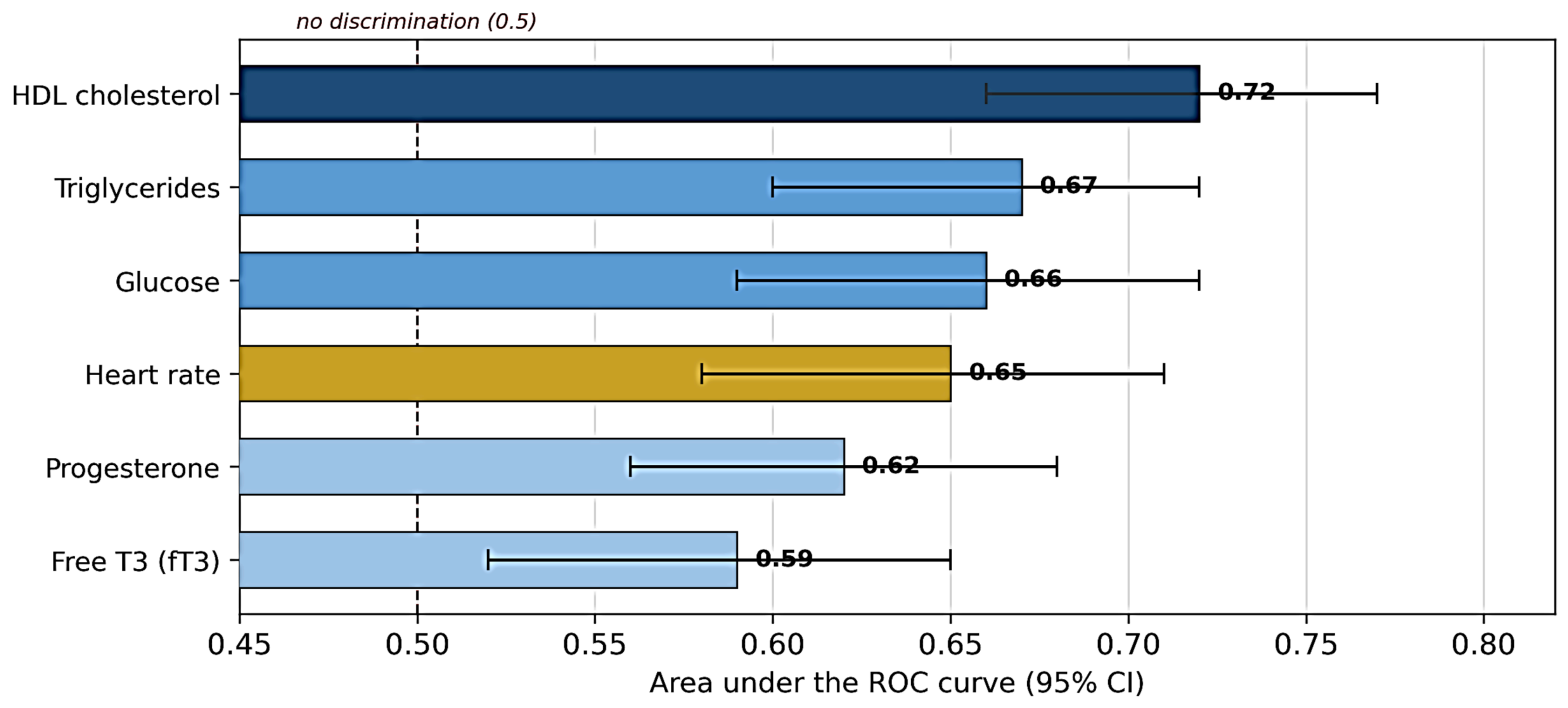
Discrimination of elevated stress (area under the ROC curve, 95% CI) for biological markers within healthcare workers.

Within healthcare workers, several biological markers showed modest discriminatory ability for elevated stress in univariable ROC analyses (Table 3, Figure 2). HDL cholesterol demonstrated the highest AUC (0.72), followed by triglycerides (0.67) and glucose (0.66), whereas heart rate, progesterone and fT3 showed weaker discrimination.

In the hierarchical multivariate logistic-regression model adjusted for age, sex, smoking and thyroid medication use, none of the investigated biomarkers remained independently associated with elevated stress. The final model showed acceptable calibration (Hosmer–Lemeshow *p* = 0.233), but limited discriminatory ability (AUC = 0.546, 95% CI 0.481–0.610) (Table 4).

## 4. Discussion

In this hospital workforce, in which nurses were the largest group, routine metabolic markers correlated only weakly with self-reported distress and burnout, yet within healthcare workers a recognisable metabolic signature—low HDL cholesterol with raised triglycerides and glucose—distinguished those reporting elevated stress. In unadjusted analyses, a faster resting heart rate and higher free triiodothyronine were associated with elevated stress; however, these associations did not persist after adjustment for age, sex, smoking and thyroid medication use. After adjustment for age, sex, smoking status and thyroid medication use, none of the investigated biomarkers remained independently associated with elevated stress. This finding suggests that the associations observed in the unadjusted analyses were attenuated after accounting for clinically relevant confounders and should therefore be interpreted with caution. These findings do not exclude a potential role of these biomarkers in occupational stress but indicate that their independent predictive value requires confirmation in larger prospective studies with external validation.

Between groups, healthcare workers had more favourable lipids and glucose than non-healthcare workers, a difference largely attributable to their younger age. The comparison between healthcare and non-healthcare workers should be interpreted cautiously because the groups differed substantially in age, work experience and other baseline characteristics. Therefore, these unadjusted differences cannot be attributed solely to occupational group.

Our findings are consistent with evidence linking chronic work stress and burnout to dyslipidaemia, impaired glucose homeostasis and a higher risk of type 2 diabetes and metabolic syndrome [10,11,12,13,14,15,16,17,23]. The inverse association of HDL cholesterol with stress echoes its established role as a cardioprotective lipid [24,25], and the triglyceride–glucose pattern is compatible with stress-related insulin resistance [25,26]. The unadjusted associations of heart rate and fT3 with elevated stress, although not independent of confounders, are nonetheless biologically plausible: a higher resting heart rate is consistent with sympathetic predominance under sustained stress, while a higher fT3 may reflect early hypothalamic–pituitary–thyroid activation rather than the suppression seen in prolonged illness [18].

The generally weak cross-sectional correlations also mirror the heterogeneity reported in the literature, where simple bivariate associations between metabolic markers and psychological symptoms are often modest. The areas under the curve in our analysis were modest to moderate (0.59–0.72), indicating that no single marker discriminates elevated stress with sufficient accuracy to serve as a standalone screening test. Although several biomarkers demonstrated statistically significant discriminatory ability, their overall performance was modest. Because the ROC-derived cut-off values were established and evaluated in the same dataset without internal or external validation, they should be regarded as exploratory. Further validation in independent cohorts is required before these findings can be translated into routine occupational-health practice.

We therefore regard these markers as candidate, rather than validated, biological indicators and as potential adjuncts to validated psychological instruments rather than substitutes for them; their value, if any, is likely to lie in a combined, multi-marker or marker-plus-questionnaire approach that would itself require prospective validation. Given the cross-sectional design, the unmeasured confounders and the absence of internal validation, these findings should be interpreted as preliminary and hypothesis-generating, and require confirmation in prospective, multicentre studies before any of these markers could be considered for routine occupational-health screening.

Mechanistically, sustained activation of the HPA axis and sympathetic nervous system can raise glucose, lower HDL cholesterol and increase triglycerides and heart rate, so these markers may reflect the cumulative physiological load of chronic stress [6,8,16]. However, because none of the markers was independently associated with stress after adjustment, the present data do not support their use for occupational-health screening, and any such application would require prospective validation.

### Strengths and Limitations

Strengths include a relatively large sample with concurrent biological and psychological assessment, standardised laboratory methods, a non-healthcare comparison group and the use of both multivariable regression and ROC analysis. The main limitations are the single-centre, cross-sectional design, which precludes causal inference; a single fasting measurement that cannot capture biological variability; reliance on self-report psychological scales; unequal group sizes; a probable healthy-worker effect; and the fact that several potential confounders were not adjusted for in the biological-marker models.

Body mass index, physical activity, diet and lipid-lowering (statin) use were not recorded. Smoking, alcohol consumption, antihypertensive, thyroid and sex-hormone medication use, work schedule (shift versus daytime work), workplace type and years of service were recorded in the study questionnaire but were not entered as covariates in these models; residual confounding by these factors therefore cannot be excluded and is a priority for future analyses. Because unrecognised thyroid disorders can directly raise fT3, the fT3 association in particular should be interpreted with caution.

Because the design is cross-sectional, our findings establish associations rather than causal, predictive or diagnostic relationships, and the markers should be regarded as candidate indicators rather than validated biomarkers. The study used a convenience sample recruited by open invitation, without an a priori sample-size calculation, which may introduce selection bias. The logistic-regression model and the ROC cut-offs were derived and evaluated in a single sample without internal validation (bootstrapping or cross-validation) or independent replication, so the reported discrimination and cut-offs are exploratory and may be optimistic. The sample was not separately powered for individual professions, so profession-specific effect estimates should be interpreted with caution and merit dedicated study. Multiple comparisons also warrant cautious interpretation, and the preliminary heart rate and fT3 associations require independent confirmation in larger, prospective, multicentre studies.

As no a priori power calculation was performed, the possibility that the study was insufficiently powered to detect small associations cannot be excluded. Since participation was voluntary, employees who were more health-conscious, available during recruitment, or willing to undergo psychological and laboratory assessments may have been more likely to participate. On the other hand, employees who experienced severe stress, sick leave, or heavy workloads may have been underrepresented. Therefore, selection bias and a healthy-worker effect cannot be ruled out.

Because multiple biomarkers and psychological outcomes were examined without formal multiplicity correction, some statistically significant findings may represent type I error and require independent confirmation. The predominance of women, the relatively healthy employed population and recruitment from a single Croatian university hospital limit external validity. The findings may not generalise to other healthcare systems, primary-care or private-sector settings, profession-specific populations, predominantly male workforces or employees absent from work because of severe stress or illness.

## 5. Conclusions

In a relatively healthy hospital workforce, routine metabolic markers showed limited direct correlation with self-reported distress. Among healthcare workers, HDL cholesterol, triglycerides and glucose demonstrated modest discriminatory ability for elevated stress in univariable ROC analyses. However, after adjustment for age, sex, smoking status and thyroid medication use, none of the investigated biomarkers remained independently associated with elevated stress. These findings suggest that routinely available biological markers may have limited value as standalone indicators of occupational stress and should be considered exploratory. Prospective, longitudinal studies with larger and more representative cohorts are needed to validate these associations, assess their temporal relationships, and determine whether the investigated biomarkers provide incremental value beyond established psychological assessment tools before any clinical or occupational-health application can be considered.

## Figures and Tables

**Table 1:** Characteristics of healthcare and non-healthcare workers.

Variable	Healthcare (n = 239)	Non-Healthcare (n = 61)	*p* *
Female sex, n (%)	191 (80)	45 (74)	0.30
Age (years)	36 (28–46)	47 (40–55)	<0.001
Total work experience (years)	15 (8–25)	27 (19–36)	<0.001
Current-post tenure (years)	8 (3–20)	20 (6–31)	<0.001
With children, n (%)	85 (36)	50 (82)	<0.001

Data are median (IQR) unless stated. * Mann–Whitney U test or chi-squared test.

**Table 2:** Metabolic and related biochemical markers in healthcare and non-healthcare workers.

Marker	Healthcare (n = 239)	Non-Healthcare (n = 61)	Median Difference (95% CI)	*p* *
Glucose (mmol/L)	4.9 (4.6–5.4)	5.2 (4.9–5.7)	0.3 (0.1 to 0.5)	0.002
Total cholesterol (mmol/L)	4.8 (4.3–5.4)	5.2 (4.6–6.0)	0.4 (0.2 to 0.7)	0.003
Triglycerides (mmol/L)	0.90 (0.63–1.40)	0.90 (0.78–1.43)	0.1 (−0.1 to 0.2)	0.28
HDL cholesterol (mmol/L)	1.4 (1.2–1.6)	1.3 (1.1–1.6)	−0.1 (−0.2 to 0.0)	0.14
LDL cholesterol (mmol/L)	3.1 (2.5–3.6)	3.7 (2.8–4.3)	0.4 (0.2 to 0.7)	0.001
Free triiodothyronine (pmol/L)	4.8 (4.5–5.3)	4.6 (4.3–4.9)	−0.25 (−0.4 to −0.1)	0.001
Cortisol (nmol/L)	304 (231–386)	296 (215–404)	−7 (−41 to 26)	0.69

Data are median (IQR). * Mann–Whitney U test. CI, confidence interval; HDL, high-density lipoprotein; LDL, low-density lipoprotein.

**Table 3:** ROC analysis of biological markers for elevated stress within healthcare workers.

Marker	AUC (95% CI)	Sensitivity (%)	Specificity (%)	Cut-Off	Youden Index	*p*
HDL cholesterol (mmol/L)	0.72 (0.66–0.77)	75	62	≤1.3	0.371	0.001
Triglycerides (mmol/L)	0.67 (0.60–0.72)	40	92	>1.9	0.322	0.03
Glucose (mmol/L)	0.66 (0.59–0.72)	55	71	>5.2	0.262	0.01
Heart rate (beats/min)	0.65 (0.58–0.71)	40	86	>87	0.258	0.03
Progesterone (nmol/L)	0.62 (0.56–0.68)	90	35	≤1.6	0.252	0.003
Free triiodothyronine (pmol/L)	0.59 (0.52–0.65)	90	34	>4.6	0.238	0.16

Cut-off values and corresponding Youden indices are exploratory, derived from the same sample without internal or external validation, and should not be interpreted as clinically established thresholds. Elevated stress defined as a DASS stress score ≥ 19. AUC, area under the curve; CI, confidence interval.

**Table 4:** Hierarchical multivariate logistic-regression analysis for elevated stress among healthcare workers.

Variable	Model 1 OR (95% CI)	*p* Value	Model 2 OR (95% CI)	*p* Value
Age (per year)	1.006 (0.98–1.03)	0.62	1.007 (0.98–1.03)	0.60
Female sex	1.007 (0.51–1.99)	0.98	1.176 (0.55–2.51)	0.68
Thyroid medication	0.566 (0.25–1.28)	0.17	0.590 (0.25–1.40)	0.23
Smoking	0.904 (0.51–1.60)	0.73	0.953 (0.53–1.70)	0.87
Heart rate (per beat/min)	-	-	1.002 (0.36–3.03)	0.89
HDL cholesterol (per mmol/L)	-	-	1.043 (0.36–3.03)	0.94
Triglycerides (per mmol/L)	-	-	1.235 (0.80–1.92)	0.35
Free triiodothyronine (per pmol/L)	-	-	1.292 (0.88–1.90)	0.19

CI—confidence interval; OR—odds ratio: Model 1: adjusted for age, sex, smoking and thyroid medication use. Model 2: Model 1 plus heart rate, HDL cholesterol, triglycerides and free triiodothyronine (fT3). Model performance (Model 2): Omnibus χ^2^ = 4.88, *p* = 0.770; Hosmer–Lemeshow χ^2^ = 10.47, *p* = 0.233; Nagelkerke R^2^ = 0.028; AUC = 0.546 (95% CI 0.481–0.610); sensitivity = 33.3%; specificity = 81.1%.

## Data Availability

The data presented in this study are available from the corresponding author upon reasonable request. The data are not publicly available owing to ethical and privacy restrictions.

## Public Involvement Statement

No public involvement in any aspect of this research.

## Guidelines and Standards Statement

This manuscript was drafted in accordance with the STROBE (Strengthening the Reporting of Observational Studies in Epidemiology) guidelines for reporting cross-sectional observational research.

## Abbreviations

The following abbreviations are used in this manuscript:

AUC	Area Under the Curve
CI	Confidence Interval
DASS-42	Depression Anxiety Stress Scales (42-item version)
fT3	Free Triiodothyronine
fT4	Free Thyroxine
HDL	High-Density Lipoprotein
HPA	Hypothalamic–Pituitary–Adrenal
IQR	Interquartile Range
LDL	Low-Density Lipoprotein
MBI-HSS	Maslach Burnout Inventory–Human Services Survey
OR	Odds Ratio
ROC	Receiver Operating Characteristic
TSH	Thyroid-Stimulating Hormone
